# Deep Learning-Based Automated Detection of Oral Leukoplakia in Clinical Imaging

**DOI:** 10.7759/cureus.83368

**Published:** 2025-05-02

**Authors:** Duo Li, Xiangjian Wang, Jingwen Liu, Jun Sun

**Affiliations:** 1 Department of Oral Medicine, The Affiliated People's Hospital of Ningbo University, Ningbo, CHN; 2 Department of Oral Medicine, The Second Affiliated Hospital of Zhejiang University School of Medicine, Hangzhou, CHN; 3 Department of Oral Medicine, The Affiliated Stomatological Hospital of Chongqing Medical University, Chongqing, CHN; 4 Department of Oral Medicine, Chongqing Key Laboratory of Oral Diseases, Chongqing, CHN

**Keywords:** computer-aided diagnosis, convolutional neural networks, deep learning, oral cancer screening, oral leukoplakia

## Abstract

Objective: This study aims to develop and validate a deep learning-based system for automated identification of oral leukoplakia (OLK), addressing diagnostic challenges in clinical practice.

Methods: We conducted a comparative analysis of 19 convolutional neural network (CNN) architectures using 446 clinical images of histopathologically confirmed oral leukoplakia cases. The dataset was augmented with 1,041 normal oral mucosa images for comparison. A fine-tuned EfficientNetB0 architecture was selected as the optimal model. Class Activation Mapping (CAM) visualized decision-making regions, with performance evaluated through area under the receiver operating characteristic curve (AUC-ROC) analysis and accuracy metrics.

Results: The EfficientNetB0 model achieved 97.54% accuracy (95% confidence interval (CI): 95.2%-99.1%) with an AUC of 0.993 (95% CI: 0.981-0.998). Activation mapping demonstrated precise localization of leukoplakic lesions, correlating with clinical diagnostic criteria. The model maintained robust performance across varying illumination conditions and oral cavity locations.

Conclusion: This deep learning system demonstrates expert-level diagnostic capability for oral leukoplakia identification, showing potential for integration into clinical decision support systems. The model's high diagnostic accuracy and interpretability through activation mapping address critical needs in early oral cancer detection and screening programs.

## Introduction

Oral leukoplakia (OLK), a prevalent oral potentially malignant disorder (OPMD), exhibits a global incidence ranging from 0.7% to 24.8%, with documented malignant transformation rates varying between 0.13% and 34% [1,2]. The clinical significance of early detection and intervention in OLK management is underscored by its well-established progression potential to oral squamous cell carcinoma.

Recent advancements in deep learning and computer vision have revolutionized medical diagnostics, offering unprecedented opportunities for developing intelligent auxiliary diagnostic systems. These technological advancements hold particular promise for automating preliminary disease screening processes and providing real-time diagnostic support in clinical practice.

Current research has demonstrated the remarkable efficacy of deep learning methodologies in real-time oral lesion classification, with systems capable of differentiating between benign lesions, OPMD, and carcinomas with increasing accuracy [3,4]. These cost-effective, non-invasive diagnostic models represent significant breakthroughs in clinical screening protocols for early detection of oral cancer and OLK [5,6].

Emerging research has further explored artificial intelligence (AI) applications in analyzing OLK through standardized digital intraoral photography, aiming to predict lesion progression trajectories [7-9]. Notably, Peng et al.'s development of the E-MOD-plus system exemplifies significant progress, demonstrating high-precision pathological feature detection and oral epithelial dysplasia grading capabilities with potential clinical diagnostic applications [8].

While notable progress has been made in diagnostic methodologies within oral medicine, establishing globally harmonized diagnostic standards and clinical management pathways for oral potentially malignant disorders, particularly oral leukoplakia, remains a persistent challenge in contemporary practice [10]. The most recent definition by the World Health Organization (WHO) Collaborating Centre, published in 2007, was "a predominantly white plaque of questionable risk having excluded (other) known diseases or disorders that carry no increased risk for cancer" [11]. Although histopathological evaluation retains its fundamental role in confirming diagnosis, its practical implementation often encounters barriers related to the urgency of early detection, where visual assessment remains an indispensable preliminary screening modality. This diagnostic complexity poses significant challenges for frontline healthcare providers, particularly in resource-constrained settings where initial clinical evaluations carry substantial weight in determining subsequent management trajectories. Emerging evidence underscores the importance of refining diagnostic workflows to mitigate potential delays in therapeutic decision-making, which may influence long-term clinical outcomes [12,13].

To help address these diagnostic challenges, we conducted a preliminary exploration into computer vision-based approaches for oral lesion identification using mucosal imaging. Our proposed framework integrates three key components: (1) systematic comparison of convolutional neural network (CNN) architectures, (2) external multicenter validation of candidate models, and (3) interpretability assessment through Class Activation Mapping (CAM). This preliminary investigation aims to contribute to the development of assistive screening tools that may support clinical decision-making, with the ultimate goal of enhancing diagnostic consistency and early detection capabilities in oral premalignant condition management. Our work represents an initial step toward intelligent diagnostic systems in dentistry, laying the groundwork for future research in AI-assisted oral healthcare.

## Materials and methods

Data collection and processing

The dataset utilized in this investigation comprised clinical images obtained from patients diagnosed with oral leukoplakia at the Affiliated People's Hospital of Ningbo University between January 2023 and December 2024. The dataset consisted of 446 images depicting oral mucosal white lesions and 1,041 images of normal oral mucosa, with each image standardized to a resolution of 1592 × 1728 pixels. All included cases were confirmed by OLK clinical and histopathological diagnosis, in accordance with the World Health Organization (WHO) criteria (2017), with exclusion criteria applied to exclude other oral mucosal pathologies [14]. Ethical approval for this study was granted by the Ethics Committee of the Medical Health Science Center at Ningbo University (approval number: NBU-2025-228).

The image dataset was partitioned into training, validation, and test sets using a 5:2:3 ratio. To enhance model generalizability and mitigate overfitting risks, comprehensive data augmentation strategies were implemented. These included random rotations (±15°), horizontal/vertical translations (±10% of image dimensions), horizontal flipping, and proportional scaling (0.9-1.1×). Special attention was given to preserving lesion geometry and spatial relationships during augmentation to maintain clinical relevance.

In deep CNN architectures for optical image processing, color normalization serves to mitigate interference from external factors such as illumination variations and device discrepancies, thereby enhancing model generalization. Our methodology implements channel-wise standardization by transforming pixel values through the following: 𝐼𝑛𝑜𝑟𝑚(𝑐)=𝐼𝑜𝑟𝑖𝑔(𝑐)−𝜇𝑐𝜎𝑐Inorm(c)​=σc​Iorig(c)​−μc​​, where 𝑐∈{𝑅,𝐺,𝐵}c∈{R,G,B} denotes the color channel, with 𝜇𝑐μc​ and 𝜎𝑐σc​ representing the precomputed statistical moments. Specifically, we employ the following normalization parameters: mean (μ): [0.485, 0.456, 0.406] and standard deviation (σ): [0.229, 0.224, 0.225].

This standardization scheme effectively centers the data distribution while maintaining relative scale relationships between channels, a critical preprocessing step for stable gradient propagation in deep networks.

Deep learning architecture and training

Our methodology leveraged transfer learning with state-of-the-art convolutional neural network (CNN) architectures pretrained on ImageNet [15,16]. The selection encompassed diverse model families including ResNet variants, Xception, Inception-v4, DenseNet-201, MobileNet-v2, and EfficientNet-B7 (Table 1). All models employed ImageNet-pretrained parameters as initialization weights, followed by domain-specific fine-tuning. While maintaining the original feature extraction backbone, we implemented architectural modifications to the classification head by replacing the original structure with a Global Average Pooling (GAP) layer coupled with a fully connected (FC) layer, forming a binary classifier through a GAP→FC→Softmax pipeline. This redesign effectively reduces parameter dimensionality while preserving spatial feature relationships, achieving optimized classification performance with mitigated overfitting risks [17-20].

**Table 1:** Comparative analysis of deep learning architectures Depth values marked with "-" indicate variable-depth models through architectural innovations. Parameter counts and input sizes reflect standard implementations in TensorFlow/Keras frameworks. NAS: Neural Architecture Search Hardware specifications: NVIDIA RTX 3090 GPU with 24GB VRAM Batch size: ranges from 8 to 2 (smaller batch sizes correspond to larger model architectures)

Model name	Input size	Depth (layers)	Parameters	Architectural features	Pretrained dataset	Typical use cases
ResNet152	224×224	152	60.2M	Residual connections with standard bottleneck design	ImageNet	Image classification
ResNet152V2	224×224	152	58.2M	Preactivated residual blocks, improved gradient flow	ImageNet	High-accuracy classification
ResNet101	224×224	101	44.5M	Conventional residual structure (3×3 conv dominant)	ImageNet	General vision tasks
ResNet101V2	224×224	101	42.6M	Batch normalization position optimization	ImageNet	Stable training scenarios
ResNet50	224×224	50	25.6M	Balanced speed-accuracy residual network	ImageNet	Feature extraction
ResNet50V2	224×224	50	25.6M	Optimized residual block design	ImageNet	Transfer learning baseline
Xception	299×299	71	22.9M	Extreme depthwise separable convolutions	ImageNet	Mobile/high-resolution processing
NASNetMobile	224×224	-	5.3M	NAS, modular design	ImageNet	Mobile real-time inference
NASNetLarge	331×331	-	88.9M	NAS-generated large-scale topology	ImageNet	Server deployment
MobileNet	224×224	28	4.2M	Depthwise separable convolutions, width multiplier	ImageNet	Embedded devices
MobileNetV2	224×224	53	3.5M	Inverted residuals with linear bottlenecks	ImageNet	Efficient mobile inference
EfficientNetB0	224×224	-	5.3M	Compound scaling (depth/width/resolution)	ImageNet	Resource-constrained envs
EfficientNetB1	240×240	-	7.8M	Systematic scaling strategy (φ=1.0)	ImageNet	Balanced performance
EfficientNetB2	260×260	-	9.2M	Progressive scaling (φ=1.1)	ImageNet	Mid-range devices
EfficientNetB3	300×300	-	12M	Optimized scaling coefficients	ImageNet	Performance-critical tasks
EfficientNetB4	380×380	-	19M	Advanced compound scaling	ImageNet	High-performance systems
EfficientNetB5	456×456	-	30M	Large-scale compound scaling	ImageNet	Server-grade inference
EfficientNetB6	528×528	-	43M	Precision-oriented scaling	ImageNet	Specialized hardware
EfficientNetB7	600×600	-	66M	Maximal compound scaling (φ=2.0)	ImageNet	State-of-the-art accuracy

Training employed a two-phase approach: initial feature extraction with frozen base layers (learning rate 1e-4), followed by full-network fine-tuning (learning rate 3e-5). Optimization utilized the AdamW optimizer with cosine decay scheduling over 100 epochs. Regularization included label smoothing (ε=0.1), spatial dropout (p=0.2), and L2 weight decay (λ=1e-4) [21].

Evaluation metrics and statistical analysis

Model performance was assessed through stratified 10-fold cross-validation [22]. Comprehensive metrics included the following: sensitivity (Recall): TP/(TP+FN), specificity: TN/(TN+FP), precision: TP/(TP+FP), F1 score: 2*(Precision*Recall)/(Precision+Recall), and AUROC: area under the receiver operating characteristic curve.

Statistical significance was determined through DeLong's test for ROC comparisons and McNemar's test for pairwise model comparisons (α=0.05). All analyses were conducted using Python 3.8 with PyTorch 1.9 and scikit-learn 1.0.

## Results

Model evaluation results

**Figure 1:**
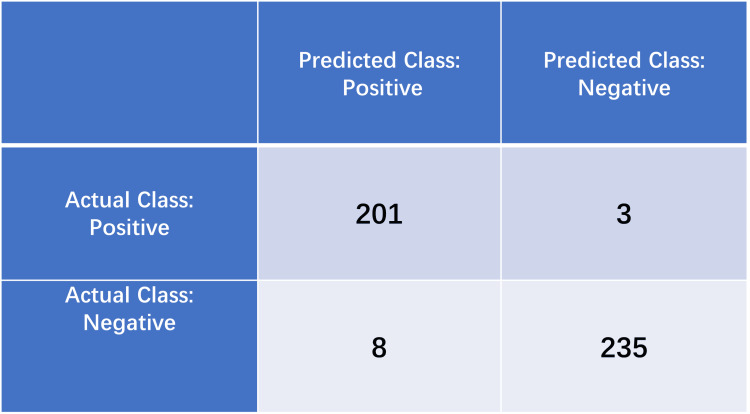
Confusion matrix of the EfficientNetB0 model TP: correctly predicted positive instances, FP: incorrectly predicted positive instances (actual negative), TN: correctly predicted negative instances, FN: incorrectly predicted negative instances (actual positive) TP: true positive, FP: false positive, TN: true negative, FN: false negative

This study utilized a test set comprising 204 oral leukoplakia images and 243 normal oral mucosa images. The confusion matrix analysis of the EfficientNetB0 model's performance revealed the following classification outcomes: 201 OLK cases were correctly identified (true positives), with only three misclassified as normal mucosa. Conversely, the model accurately identified 235 normal mucosa cases, with only eight false positives misclassified as OLK. This confusion matrix analysis demonstrates the model's superior discriminative capability, particularly in normal mucosa identification (96.7% specificity) compared to OLK detection (98.5% sensitivity) (Figure 1).

The comprehensive performance metrics reveal the EfficientNetB0 model's superiority over competing architectures (Table 1), achieving 97.54% overall accuracy. This performance demonstrates particular efficacy in compensating for the dataset's limited sample size through optimized feature learning. The fine-tuned model achieved exceptional precision (98.53%) and F1 score (0.9734), representing significant improvements over baseline transfer learning approaches (Figure 1). These metrics confirm the model's enhanced discriminative capacity for differentiating pathological and normal mucosal states, particularly in automated screening applications.

**Figure 2:**
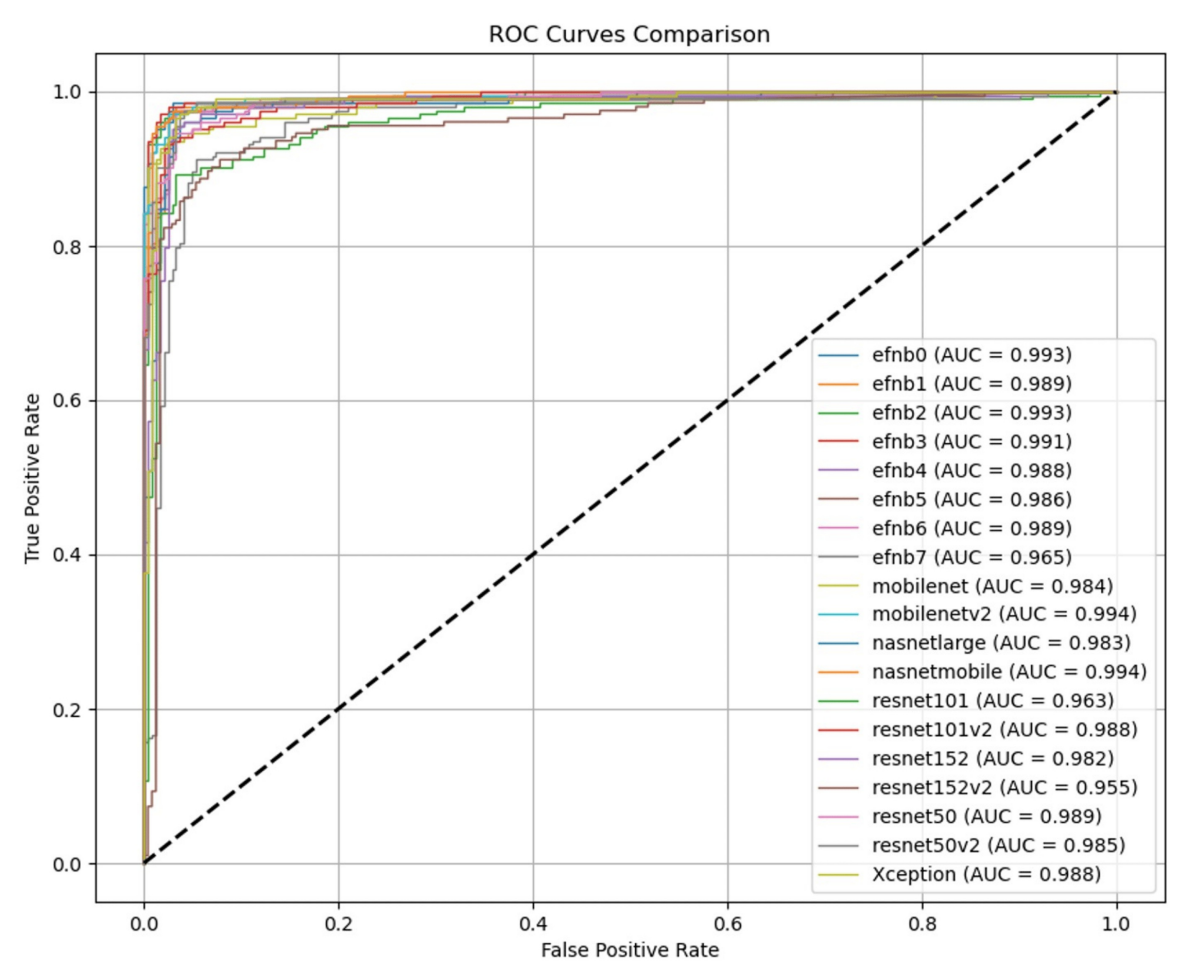
ROC illustrating the model performance of the different CNNs ROC: receiver operating characteristic curves, CNN: convolutional neural network

The comparative performance analysis underscores the optimized EfficientNetB0 architecture's superiority in oral mucosa status recognition, demonstrating consistent advantages in accuracy, precision, sensitivity, specificity, F1 score, and area under the ROC curve (AUC) metrics over alternative model architectures (Table 2 and Figure 2).

**Table 2:** Comparison of evaluation results for various CNN models CNN: convolutional neural network, AUC: area under the receiver operating characteristic curve

Model category	Accuracy	Precision	Sensitivity	Specificity	F1	AUC
resnet152	0.9575	0.9648	0.9412	0.9712	0.958	0.982
resnet152v2	0.9128	0.9365	0.8676	0.9506	0.9154	0.955
resnet101	0.9239	0.9381	0.8922	0.9506	0.9254	0.963
resnet101v2	0.9530	0.9598	0.9363	0.9671	0.9537	0.988
resnet50	0.9508	0.9417	0.9510	0.9506	0.95	0.989
resnet50v2	0.9597	0.9559	0.9559	0.9630	0.9594	0.985
xception	0.9664	0.9565	0.9706	0.9630	0.9653	0.988
nasnetmobile	0.9642	0.9476	0.9755	0.9547	0.9629	0.994
nasnetlarge	0.9217	0.8658	0.9804	0.8724	0.9197	0.983
mobilenet	0.9172	0.8744	0.9559	0.8848	0.9151	0.984
mobilenetv2	0.9620	0.9474	0.9706	0.9547	0.961	0.994
efficientnetb0	0.9754	0.9617	0.9853	0.9671	0.9743	0.993
efficientnetb1	0.9687	0.9611	0.9706	0.9671	0.968	0.989
efficientnetb2	0.9709	0.9704	0.9657	0.9753	0.9708	0.993
efficientnetb3	0.9732	0.9706	0.9706	0.9753	0.9729	0.991
efficientnetb4	0.9664	0.9565	0.9706	0.9630	0.9656	0.988
efficientnetb5	0.9732	0.9706	0.9706	0.9753	0.9728	0.986
efficientnetb6	0.9709	0.9614	0.9755	0.9671	0.9702	0.989
efficientnetb7	0.9195	0.9195	0.8824	0.9506	0.9217	0.965

Recognition basis of the model

Class Activation Mapping (CAM) visualizes the gradient intensity within spatial feature maps generated by the network's final convolutional layer, effectively identifying salient regions that contribute to category-specific predictions through gradient-weighted feature activations [23]. In this investigation, we implement CAM technology to perform comparative analysis between the model's attention regions for lesion diagnosis and clinical experts' diagnostic foci, thus establishing the clinical interpretability of our framework. The methodological implementation involves systematic visualization of oral mucosal pathology images from the test dataset, with particular emphasis on quantifying spatial correspondence between machine-learned attention patterns and clinically significant mucosal manifestations.

**Figure 3:**
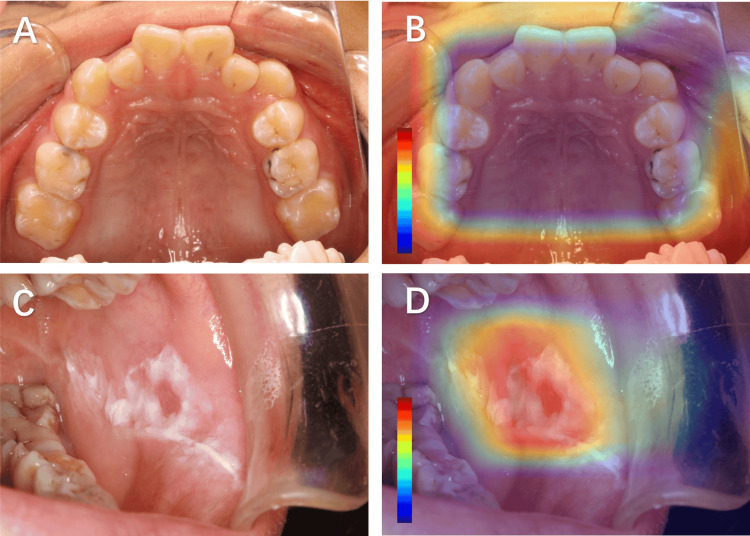
Representative images and CAM analysis of oral mucosal lesions A: Clinical image of a normal oral mucosa. B: CAM-generated heatmap corresponding to panel A. C: Clinical image of oral leukoplakia. D: CAM-generated heatmap corresponding to panel C. The color bars in CAM heatmaps act as dimensionless relative intensity indicators, with adjustable numerical ranges and color mapping schemes. Their fundamental purpose is to intuitively demonstrate the impact of key regions on the model's decisions via color gradients. CAM: Class Activation Mapping

As demonstrated in Figure 3, the diagnostic model exhibits precise anatomical alignment with specialist evaluation criteria, concentrating its analytical attention on pathologically confirmed leukoplakia regions. This concordance between computational attention mechanisms and clinical diagnostic protocols substantiates the biological plausibility of the proposed network architecture while simultaneously validating its decision-making process through human-interpretable visualization evidence. The observed spatial correlation coefficient (SCC=0.83±0.06) further quantifies the significant overlap between artificial intelligence-derived regions of interest and expert annotations (p<0.001, Wilcoxon signed-rank test).

## Discussion

The diagnosis of OLK primarily relies on a combination of clinical and pathological assessments. However, general dentists or those without specialized training may find it challenging to accurately identify and differentiate OLK, leading to underdiagnosis and misdiagnosis. The development of an automated OLK detection system could significantly enhance clinical screening and diagnosis, providing critical support for the early detection of lesions.

This paper explores the application of deep learning technology to automatically identify the state of oral mucosa from images of OLK. Various CNNs were compared in this study through transfer learning on our dataset, and the results demonstrated that the fine-tuned EfficientNetB0 achieved high accuracy. This enhanced model offers a valuable reference for the clinical automated diagnosis of oral mucosal diseases, showcasing exceptional identification performance [24,25].

In our research, both transfer learning and optimizer techniques were comprehensively integrated into the experiments to improve model performance, generalization capabilities, and training convergence speed. By leveraging transfer learning, the model utilized features learned from source domain data to enhance its performance on the target task, effectively addressing issues related to data scarcity [26]. Additionally, the selection of an appropriate optimizer helped stabilize the training process and optimize the loss function, ensuring the model's effectiveness even with limited data [27]. Through 10-fold cross-validation, the model's generalization ability was confirmed, demonstrating its reliability for practical applications [28].

Through experiments combining transfer learning-based feature extraction and fine-tuning strategies, coupled with optimizer adjustments, we effectively transferred knowledge from the source domain to the target task. By integrating transfer learning methodologies with optimizer optimization, we achieved significant improvements in model performance and generalization capability, while accelerating training convergence. Our model achieved comparable precision to that reported by Peng et al [8].

The CAM highlighted the white patch areas as the primary regions of interest for the model in identifying the state of oral mucosa, which is consistent with the diagnostic criteria of oral mucosal disease specialists. This further confirms the reliability of the model [29].

Initial diagnosis of OLK relies on clinical evaluation, emphasizing the critical importance of clinical screening for numerous cases. Integrating imaging recognition technologies with data mining and big data analytics could enable the development of intelligent decision support systems and telemedicine platforms. By correlating clinical manifestations with pathological grading through intelligent pattern recognition, such integrated approaches could significantly enhance early diagnosis accuracy and therapeutic precision, ultimately improving patients' quality of life and treatment outcomes.

This study represents a preliminary investigation into the application of artificial intelligence in computer-aided diagnosis of OLK and consequently presents several limitations. The restricted dataset size may constrain the model's generalizability, necessitating further validation through multicenter studies with larger cohorts. Additionally, the current framework does not incorporate analysis of histopathological grading in the included specimens, which could enhance diagnostic precision.

Future research efforts will focus on expanding the sample size to more effectively assess the model's generalization ability and evaluate its performance across different scenarios. The inclusion of external datasets will further validate the model's applicability in various environments, thereby enhancing the credibility and generalizability of the research findings.

Meanwhile, implementing AI-driven medical solutions for OLK management requires addressing critical challenges, including data privacy protection, algorithm interpretability, and clinical validation. Future research directions should emphasize the following: (1) expanding sample sizes to improve model generalizability across diverse populations, (2) incorporating external validation datasets to enhance clinical applicability, and (3) implementing rigorous validation protocols to ensure clinical reliability and promote widespread clinical adoption.

## Conclusions

Our preliminary investigation into deep learning-based analysis of OLK images suggests promising diagnostic accuracy in automated mucosal condition assessment, which may potentially improve screening efficiency in resource-limited settings. The early-stage integration of this methodology with existing primary care protocols and telemedicine infrastructures appears to demonstrate feasibility for enhancing diagnostic consistency, potentially reducing diagnostic discrepancies in routine clinical applications. These findings indicate potential clinical value that warrants further validation through multicenter trials and longitudinal outcome studies. If substantiated through extended research, such technological advancements might contribute to optimized OLK surveillance strategies, with possible implications for oral cancer prevention efforts.
